# Effect of Weight Loss on Serum Osteocalcin and Its Association with Serum Adipokines

**DOI:** 10.1155/2015/508532

**Published:** 2015-02-15

**Authors:** Mohammed S. Albadah, Hafedh Dekhil, Shaffi Ahamed Shaik, Mohammed A. Alsaif, Mustafa Shogair, Shahid Nawaz, Assim A. Alfadda

**Affiliations:** ^1^Obesity Research Center, College of Medicine, King Saud University, P.O. Box 2925 (98), Riyadh 11461, Saudi Arabia; ^2^Department of Clinical Nutrition, King Khalid University Hospital, King Saud University, P.O. Box 7805 (104), Riyadh 11472, Saudi Arabia; ^3^Department of Family and Community Medicine, College of Medicine, King Saud University, P.O. Box 2925, Riyadh 11461, Saudi Arabia; ^4^Department of Community Health Sciences, College of Applied Medical Sciences, King Saud University, P.O. Box 10219, Riyadh 11433, Saudi Arabia; ^5^Department of Medicine, College of Medicine, King Saud University, P.O. Box 2925 (38), Riyadh 11461, Saudi Arabia

## Abstract

Studies have suggested that osteocalcin, a bone formation marker, is related to body metabolism and insulin sensitivity. Whether this relation is mediated through an interaction with adipokines remains unclear. The aim of this study was to assess the effect of weight loss on serum osteocalcin and its relation with three adipokines, adiponectin, chemerin, and resistin. Forty-nine obese nondiabetic males completed a four-month dietary program. Body mass index (BMI) decreased significantly from 39.7 ± 7.6 to 37.8 ± 7.6 (*P* < 0.001). This was associated with significant reduction in waist circumference, fasting blood glucose, HOMA-IR, total and LDL-cholesterol, bone-specific alkaline phosphatase (BAP), and resistin (*P* < 0.05). There was significant increase in serum adiponectin and undercarboxylated osteocalcin (uOC) (*P* < 0.001). The changes in uOC levels were negatively correlated with changes in serum triglycerides (*r* = −0.51, *P* < 0.001) and positively correlated with changes in BAP (*r* = 0.52, *P* < 0.001). In contrast, the changes in uOC were not correlated with changes in BMI, waist circumference, fasting blood glucose, HOMA-IR, total and LDL-cholesterol, hsCRP, vitamin D, and circulating adipokines. We concluded that the increase in serum uOC following weight loss is not related to the changes in circulating adipokines levels.

## 1. Introduction

The prevalence of obesity, a multifactorial disease caused by a complex interplay between genetic predisposition and the environment [1], is constantly growing posing enormous health burden on societies worldwide [2]. Obesity is associated with a variety of metabolic and hormonal dysfunctions such as the development of insulin resistance and type 2 diabetes mellitus, leading to increased morbidity and mortality in the affected subjects [3]. It is now widely accepted that, besides being an important energy depot, white adipose tissue, the predominant type of adipose tissue, is a metabolically active organ that may regulate a variety of systemic processes through the secretion of bioactive substances, collectively known as adipokines [4]. Serum adipokines concentrations are closely linked to the degree of obesity, and altered adipokines secretion is hypothesized to mediate the relationship between obesity, inflammation, insulin resistance, and cardiometabolic disease [5, 6].

Weight loss, through energy restriction and/or exercise, improves metabolic health and reduces morbidity and mortality associated with obesity [7]. More specifically, losing 5–15% of initial body weight is associated with improved plasma lipid profile, decreased blood pressure, and increased insulin sensitivity [8]. Although the mechanisms that link weight loss to disease risk reduction remain uncertain, evidence suggests adipokines may play a role [9]. For example, adiponectin is a fat-cell-derived hormone shown to be inversely related to body weight, visceral fat mass, and insulin resistance. Overweight and obese individuals with higher adiponectin levels are more likely to show a favorable metabolic and inflammatory profile and hence less likely to develop cardiovascular obesity related cardiometabolic complications [10, 11].

In recent years, it has been reported that the skeletal system also plays a role in the regulation of energy and glucose metabolism [12]. The presumed protective effect of obesity on osteoporosis has urged researchers to think about the presence of hormones that affect both the bones and the energy metabolism. The first rodent studies showed that osteocalcin (OC), which is secreted from osteoblasts, increased insulin secretion as well as insulin sensitivity. It has been shown in experimental animals that recombinant OC administration increases insulin sensitivity and adiponectin levels [13]. Lee et al. demonstrated that OC is involved in glucose metabolism by increasing insulin secretion and cell proliferation in pancreatic *β*-cells and by upregulating the expression of the adiponectin gene in adipocytes, thus improving insulin sensitivity [14, 15]. Studies in adults and children, although few in number, have shown an association between low OC levels and insulin resistance [16–18]. Recently, it has been reported that, in adults, circulating OC levels are associated with improved glucose tolerance and insulin secretion and sensitivity, independent of the plasma adiponectin level [19, 20].

Clinically, serum OC measurements and other bone makers may help in assessing fracture risk in patients with osteoporosis. They may be useful for monitoring therapy, predicting early response to therapy, and identifying noncompliance and nonresponders [21].

To further understand the complex relationship between OC and circulating adipokines, we sought to study the effect of weight loss on different forms of serum OC and its relation to three of the most widely investigated adipokines, adiponectin, chemerin, and resistin and whether the changes in circulating adipokines are significant and independent predictors of changes in circulating OC levels. To the best of our knowledge, this is the first study investigating the changes in different forms of OC in relation to circulating adipokines following weight loss.

## 2. Materials and Methods

A total of 102 consecutive obese nondiabetic male subjects were recruited from the Nutrition Clinic at King Khalid University Hospital, King Saud University (KSU). Subjects with significant liver disease, renal failure, endocrine disorders, and smoking, or taking medications that could affect body weight or vitamin K level were not included in the study. Forty-nine participants completed a four-month dietary program and were included in the final analysis. This study was performed at the Obesity Research Center, College of Medicine, Riyadh, Saudi Arabia. The Institutional Review Board approved this study and all participants gave informed consent.

To evaluate changes in clinical and biochemical parameters in response to weight loss, subjects underwent a four-month dietary control program. Subjects were given instructions folder listing different combinations of meals and snacks that collectively would provide 1,500 Kcal/day. They then used their food lists to self-select three meals and two snacks a day during the duration of the study. They were asked to consume two servings of green vegetables a day. Follow-up telephone calls were made once every two weeks to ensure compliance. Subjects were also advised at the first visit to maintain a healthy lifestyle in general, but they did not participate in any organized physical activity programs. Selected circulating adipokines, several metabolic parameters, and TOC, cOC, and uOC were measured before and after intervention. Venous blood samples were collected between 8.00 and 10.00 in the morning after an overnight fasting and then centrifuged (1000 g, 10 min, 4°C). The serum was separated, aliquoted, and stored at −80°C.

Anthropometric measures including height and weight were taken while the subject was wearing light clothing, and body mass index (BMI) was calculated. The waist circumference (WC; in cm) was obtained, using an anthropometric tape, as the minimum value between the iliac crest and the lateral costal margin. Body height (in m) was measured using a standing stadiometer. Body weight (in kg) was measured to the nearest 0.1 kg using a calibrated balance scale. BMI was calculated as body weight divided by height squared (kg/m^2^).

Serum glucose, triglycerides, total cholesterol, high-density lipoprotein (HDL) cholesterol, and bone-specific alkaline phosphatase (BAP) were determined using Dimension Xpand Plus integrated clinical chemistry autoanalyzer (Siemens Healthcare Diagnostics, USA). Low-density lipoprotein (LDL) cholesterol was calculated using Friedewald's equation [22]. High-sensitivity C-reactive protein (hsCRP) was measured using BN ProSpec Nephelometer (Siemens Healthcare Diagnostics, USA). Plasma insulin quantity was determined by electrochemiluminescence using Cobas e411 immunoanalyzer (Roche Diagnostics, USA). Insulin resistance was represented using the “homeostasis model assessment of insulin resistance” (HOMA-IR), which was determined according to the following equation: HOMA-IR = fasting plasma glucose (mmol/L) × fasting plasma insulin (mU/mL)/22.5 [23]. Serum levels of 25-OH vitamin D were determined using Cobas e 602 automatic analyzer (Roche Diagnostics, USA).

Circulating levels of adiponectin, resistin, and chemerin were determined using ELISA kits (R&D, USA). Intra- and interassay coefficients of variations (CVs) for these parameters were chemerin intraassay CV = 4.3% interassay CV = 7.3%, adiponectin intraassay CV = 7.4% and interassay CV = 8.4%, and resistin intraassay CV = 4.7% and interassay CV = 8.4%.

Serum TOC was measured using N-MID Osteocalcin kit (Elecsys, Roche diagnostic Ltd., Switzerland). The serum uOC and serum cOC were measured using EIA kits (Takara Bio Inc., Japan). The undercarboxylated EIA kit uses a set of monoclonal antibodies reactive to the uOC and less reactive to cOC at the amino acid positions 17, 21, and 24. The carboxylated EIA kit uses a set of monoclonal antibodies reactive to cOC at the amino acid position 17. The intraassay and interassay variabilities were, respectively, 5.7% and 6.2% for cOC, 10.2% and 9.8% for uOC, and 3.4% and 3.6% for TOC.

## 3. Statistical Analysis

Data were analyzed using SPSS Pc+ version 21.0 statistical software. Descriptive statistics (mean and standard deviation) were used to describe the quantitative study and outcome variables. Kolmogorov-Smirnov test was used to test the data distribution. Before the statistical analysis, a logarithmic transformation of the nonnormally distributed parameters was performed to approximate a normal distribution. Student's paired *t*-test was used to compare the difference of clinical and metabolic characteristics between baseline and 4-month time period of dietary control program. Karl Pearson correlation analysis was carried out to observe the correlation between changes in circulating uOC and uOC/TOC and changes in selected anthropometric and metabolic variables after weight loss. Multiple regression analysis was done by considering serum uOC and uOC/TOC levels as outcome variables to observe whether the changes in these variables are explained by the other independent variables (age, change in BMI, change in BAP, and change in serum triglycerides). A *P* value of <0.05 was considered as statistically significant.

## 4. Results

The mean age of 49 study subjects was 32.3 ± 8.7 years (range 20–50 years). After 4 months on a dietary control program, BMI decreased significantly from 39.7 ± 7.6 to 37.8 ± 7.6 (*P* < 0.001). There was significant reduction in waist circumference, fasting blood glucose (FBG), HOMA-IR, total and LDL-cholesterol, BAP, and resistin (*P* < 0.001, except for HOMA-IR *P* = 0.02). We found significant increase following the dietary control program in adiponectin, uOC, and uOC/TOC ratio (*P* < 0.001), whereas HDL-cholesterol, triglycerides, hsCRP, vitamin D, chemerin, TOC, and cOC were unchanged (Table 1).

**Figure 1:**
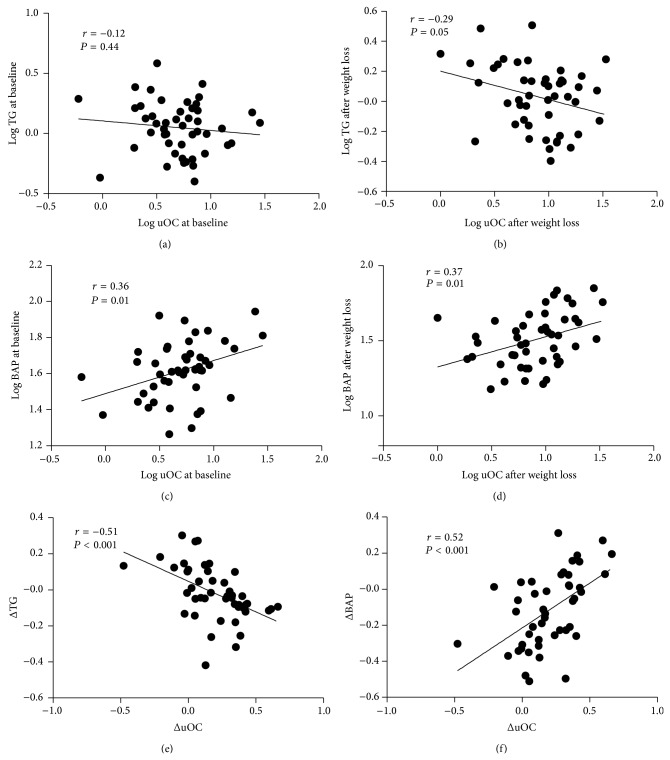
The correlation of serum undercarboxylated osteocalcin (uOC) with serum triglycerides (TG) and bone-specific alkaline phosphatase (BAP). Serum TG did not correlate with uOC at baseline (*r* = −0.12, *P* = 0.44) (a) but showed a negative correlation with uOC after weight loss (*r* = −0.29, *P* = 0.05) (b). BAP was positively correlated with uOC at baseline (*r* = 0.36, *P* = 0.01) (c) and after weight loss (*r* = 0.37, *P* = 0.01) (d). The changes in serum uOC levels were negatively correlated with changes in serum TG (*r* = −0.51, *P* < 0.001) (e) and positively correlated with changes in BAP (*r* = 0.52, *P* < 0.001) (f).

At baseline, serum TOC levels correlated negatively with fasting blood glucose (*r* = −0.43, *P* = 0.00). Serum uOC and uOC/TOC ratio were positively correlated with LDL cholesterol (*r* = 0.35, *P* = 0.02, and *r* = 0.31, *P* = 0.03, resp.). However, these associations disappeared following weight loss. Serum triglycerides did not correlate with uOC at baseline but showed a negative correlation with uOC after weight loss (*r* = −0.12, *P* = 0.44, and *r* = −0.29, *P* = 0.05; Figures 1(a) and 1(b), resp.). BAP was positively correlated with uOC at baseline and after weight loss (*r* = 0.36, *P* = 0.01, and *r* = 0.37, *P* = 0.01; Figures 1(c) and 1(d), resp.). In addition, following weight loss, uOC/TOC ratio was negatively correlated with serum triglycerides (*r* = −0.31, *P* = 0.03). There were no significant associations between cOC and all the parameters that were studied.

We then assessed the relation between changes in circulating uOC and uOC/TOC ratio and changes in serum adipokines and selected anthropometric and metabolic variables 4 months after the dietary control program. The changes in serum uOC levels were negatively correlated with changes in serum triglycerides (*r* = −0.51, *P* < 0.001, Figure 1(e)) and positively correlated with changes in BAP (*r* = 0.52, *P* < 0.001, Figure 1(f)). Similarly, the changes in the uOC/TOC ratio were negatively correlated with changes in serum triglycerides (*r* = −0.64, *P* < 0.001) and positively correlated with changes in BAP (*r* = 0.45, *P* < 0.001) (Table 2). In contrast, the changes in serum uOC and uOC/TOC ratio were not significantly correlated with changes in BMI, WC, fasting blood glucose, HOMA-IR, serum cholesterol, hsCRP, vitamin D, and circulating adipokines (Table 2).

The changes in serum uOC levels after the dietary control program were best explained by changes in serum triglycerides and BAP (*r* ^2^ = 0.52, *P* < 0.001). Similarly, the changes in uOC/TOC were best explained by changes in serum triglycerides and BAP (*r* ^2^ = 0.58, *P* < 0.001). Neither age nor changes in BMI made a unique contribution in these correlation analyses.

## 5. Discussion

This study showed significant reduction in waist circumference, insulin resistance, and serum resistin following a dietary control program. This finding is similar to other intervention studies showing a beneficial effect of weight loss on central obesity and insulin resistance [8]. Furthermore, weight loss resulted in a significant increase in serum adiponectin, uOC, and uOC/TOC ratio. Results of experimental studies on reciprocal bone-energy metabolism relationships mediated by adipokines and OC are fairly consistent. However, clinical data on the association between circulating adiponectin levels and OC are controversial [24]. Previous human studies reported a positive association between serum adiponectin and OC [25–28], whereas other studies were not able to demonstrate a significant and independent relationship [29–31]. Both elevated levels of circulating adiponectin and uOC are associated with an improvement in insulin sensitivity. Although we noticed an increase in serum adiponectin and uOC and its ratio to TOC after weight loss, we did not find these increments related to each other. Our results support recent reports which showed that circulating OC levels are associated with improved glucose tolerance and insulin secretion and sensitivity, independent of the plasma adiponectin level [20, 32].

Emerging evidence has shown that resistin, a peptide hormone classified as an adipokine although in humans it is mainly produced by mononuclear cells and macrophages, is important in regulating insulin resistance, diabetes, inflammatory processes, immunity, and bone metabolism [33, 34]. In older subjects with osteoporotic hip fracture, serum osteocalcin concentration was inversely associated with resistin and positively with leptin, leptin/resistin ratio, and adiponectin/resistin ratio. In multivariate regression models, osteocalcin was an independent predictor of serum leptin, resistin, leptin/resistin, and adiponectin/resistin ratios [24]. We noticed a significant decrease in resistin level following the dietary control program, but this reduction was not associated with the increase in uOC level. Therefore, it might be that the mechanism by which elevated uOC would cause an improvement in insulin sensitivity is not linked to circulating resistin levels. Further studies are needed to confirm this assumption.

It has been previously reported that chemerin, a novel adipokine that is expressed and secreted at the high levels by adipocytes, is also expressed and secreted but at lower level by osteoblasts [35]. Secreted chemerin activates chemerin-like receptor 1 (CMKLR1), a G-protein coupled receptor expressed by various cell types including osteoblasts [36]. Abrogation or knockdown of CMKLR1 expression increased the expression of osteoblast markers, including alkaline phosphatase, and type I collagen [37]. However, similar to other adipokines, the overall significance of chemerin to bone homeostasis and pathophysiology is currently unclear. We did not find any correlation between uOC and circulating chemerin levels at baseline or after weight loss. Furthermore, the increase in uOC noticed after weight loss was not associated with a change in circulating chemerin. Further studies will be required to investigate the relation between chemerin and bone cell function. For example, it has been shown recently that the increase in the accumulation of adipocytes and their secreted adipokines in bone marrow affects several aspects of bone remodeling [35].

Both uOC and uOC/TOC ratio increased significantly after weight loss, but there was no increase in TOC level. Levinger et al. reported that uOC, but not TOC, increased significantly following an exercise program in obese nondiabetic men [38]. The discrepancy between the changes in uOC and TOC after a dietary or an exercise program is pointing towards the fact that these two measurements might have different biological significance. It is after correcting for age and the change in BMI that 52% of the change in uOC and 58% in the change in uOC/TOC ratio were explained by changes in BAP and serum triglycerides levels (*P* < 0.001). Both OC and BAP are secreted by osteoblasts and considered as indicators of osteoblast function [39]. The positive correlation between the changes in uOC and the changes in BAP could be an indicator of the balance state of bone remodeling during the weight loss program. Furthermore, the relation between serum triglycerides and OC is supported by recent human studies which have consistently reported inverse associations between circulating osteocalcin and components of metabolic syndrome in several populations [40–42], including our own study [43]. In the present study we did not show a significant reduction in serum triglycerides level following the dietary control program. This could be explained by the relatively low triglycerides values in our patients at baseline and the small sample size. However, the modest changes in serum triglycerides level were associated with an increase in circulating uOC. This study would also help in clarifying the relationship between OC and some cardiometabolic risk factors in men. While the large study in Australian men showed an inverse association between plasma OC and metabolic syndrome which seemed to be mediated by waist circumference, hyperglycemia, and hypertriglyceridemia [44]; other studies did not find the same [45]. This contradiction could be partially explained by the reliance on the TOC rather than the uOC measurements in these studies. We here confirmed that the uOC and the uOC/TOC ratio were closely related to the change in serum triglycerides seen after weight loss and thus would be more useful than TOC measurement in assessing the relation between OC and cardiometabolic risk.

A limitation of our study is that we recruited only male subjects; therefore the findings could not be extrapolated to females. Second, we did not assess vitamin K intake which could influence OC and bone metabolism. However, we think that this did not affect our conclusions since all study subjects were maintained on a dietary program which is expected to provide the same amount of vitamin K. All participants were maintained on equal daily servings of green vegetables throughout the study period which is considered as the major source of vitamin K in food. Furthermore, no subjects were taking medications that could affect vitamin K level. On the other hand, the prospective design of this study and the measurement of all three forms of circulating OC in association with several metabolic components and adipokines provide valuable information that could add significantly to the current literature.

In conclusion, we have reported the effect of weight loss on different forms of serum OC and its relation with three adipokines, namely, adiponectin, chemerin, and resistin, in an obese male cohort. The increase in serum uOC and the uOC/TOC ratio following weight loss was not related to the change in circulating adipokines levels. The underlying mechanism by which weight loss causes an alteration in circulating OC level is most likely not related to circulating adipokines levels.

## Figures and Tables

**Table 1:** Change in clinical and metabolic characteristics 4 months after weight loss (*n* = 49).

Variable	Baseline	4 months	*P*
Age	32.29 ± 8.68		
BMI (kg/m^2^)	39.72 ± 7.58	37.83 ± 7.61	**<0.001**
WC (cm)	119.01 ± 13.76	112.6 ± 14.2	**<0.001**
FBG (mmol/L)	5.38 ± 0.71	5.03 ± 0.69	**<0.001**
HOMA-IR	5.44 ± 3.9	4.47 ± 2.76	**0.02**
Total cholesterol (mmol/L)	4.86 ± 0.88	4.44 ± 0.84	**<0.001**
LDL-cholesterol (mmol/L)	3.27 ± 0.78	2.9 ± 0.71	**<0.001**
HDL-cholesterol (mmol/L)	1.01 ± 0.23	1 ± 0.2	0.21
Triglycerides (mmol/L)	1.26 ± 0.65	1.2 ± 0.6	0.21
hsCRP (mg/L)	9.55 ± 7.81	9.33 ± 8.26	0.23
BAP (*μ*g/L)	44.3 ± 16	35 ± 14.31	**<0.001**
Vitamin D (nmol/L)	34.24 ± 25.32	38.76 ± 23.95	0.06
Adiponectin (*μ*g/mL)	25.25 ± 10.61	36.3 ± 18.07	**<0.001**
Chemerin (ng/mL)	113.76 ± 33.41	107.6 ± 54.27	0.29
Resistin (*μ*g/mL)	28.34 ± 7.29	8.02 ± 3.38	**<0.001**
Total OC (ng/mL)	19.36 ± 6.6	19.59 ± 5.7	0.18
Carboxylated OC (ng/mL)	10.64 ± 5.16	11.22 ± 5.31	0.41
Undercarboxylated OC (ng/mL)	6.44 ± 5.3	10.21 ± 7.22	**<0.001**
Undercarboxylated/total OC	0.34 ± 0.26	0.51 ± 0.29	**<0.001**

Data are presented as the mean ± standard deviation. BMI: body mass index; WC: waist circumference; FBG: fasting blood glucose; HOMA-IR: homeostasis model assessment of insulin resistance; LDL: low-density lipoprotein; HDL: high-density lipoprotein; hsCRP: high-sensitivity C-reactive protein; BAP: bone-specific alkaline phosphatase; and OC: osteocalcin. The *P* values were yielded from the baseline-versus-4-month comparison of the values for each of the measured parameters. *P* values < 0.05 (bolded) were considered statistically significant.

**Table 2:** Correlation analysis between changes in circulating undercarboxylated OC and undercarboxylated/total OC and changes in selected anthropometric and metabolic variables 4 months after dietary program.

Variable	Change in uOC		Change in uOC/total OC	
	*r*	*P*	*r*	*P*
ΔBMI (kg/m^2^)	−0.21	0.15	−0.12	0.41
ΔWC (cm)	−0.13	0.4	−0.15	0.31
ΔFBG (mmol/L)	0.23	0.13	0.17	0.24
ΔHOMA-IR	−0.01	0.93	0.03	0.82
ΔTotal cholesterol (mmol/L)	−0.18	0.23	−0.24	0.1
ΔLDL-cholesterol (mmol/L)	−0.05	0.73	−0.08	0.61
ΔHDL-cholesterol (mmol/L)	0.05	0.74	0.14	0.34
ΔTriglycerides (mmol/L)	−0.51	**<0.001**	−0.64	**<0.001**
ΔhsCRP (mg/L)	−0.09	0.55	0.09	0.53
ΔBAP (*μ*g/L)	0.52	**<0.001**	0.45	**<0.001**
ΔVitamin D (nmol/L)	−0.15	0.3	−0.09	0.53
ΔAdiponectin (*μ*g/mL)	0.07	0.64	0.1	0.5
ΔChemerin (ng/mL)	−0.1	0.5	−0.2	0.18
ΔResistin (*μ*g/mL)	−0.06	0.68	−0.13	0.41

Changes are expressed as Δ: variable at baseline minus variable measured 4 months after dietary control program.
